# Statins as early therapy to mitigate COVID-19 (SARS-CoV-2)-associated ARDS and cytokine storm syndrome – time is of the essence

**Published:** 2020-04-18

**Authors:** Narci Teoh, Geoff Farrell

**Affiliations:** Gastroenterology and Hepatology Unit, Medicine and Surgery Program, The Australian National University, Canberra, Australian Capital Territory, Australia

**Keywords:** COVID-19, SARS-CoV-2, statins, atorvastatin, acute respiratory distress syndrome, cytokine storm, microparticles, hyperinflammation, ACE 2


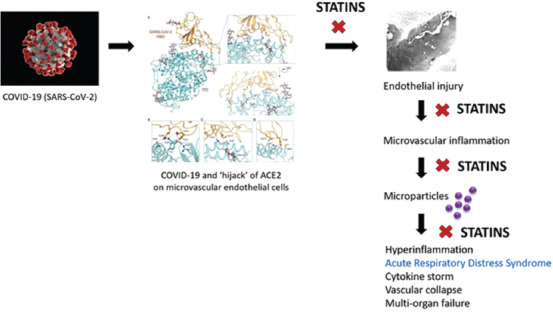


While most coronavirus disease 2019 (COVID-19) infections run a benign course with recovery, the inexorable rise in case fatalities in several countries is grim testimony to the lack of effective therapies to arrest the course of severe infections. Recent observations of Mehta *et al*. [1] and Matthay *et al*. [2] have piqued interest in the pathogenic sequence of these severe infections. This leads us to propose an early, safe, and widely available pharmacological intervention that could be used pre-emptively to avert fatal outcomes in severe acute respiratory syndrome coronavirus 2 (SARS-CoV-2)-infected patients.

In addition to older age (>65 years), obesity, and male gender, predictors of COVID-19 mortality are high C-reactive protein (CRP) (>100 mg/L in fatal vs. 3 mg/L in non-fatal), raised ferritin (mean 1297 ng/mL in non-survivors vs. 614 ng/mL in survivors), increased white blood cell count, lymphopenia, and abnormal chest imaging [1,3,4]. Hyperinflammation, cytokine storm, and acute respiratory distress syndrome (ARDS) with profound vascular collapse causing multiorgan failure have been consistently described [1-4]. COVID-19-induced hyperinflammation is characterized by increased production of tumor necrosis factor-α (TNF-α), macrophage inflammatory protein-1-α (MIP1-α), interleukin (IL)-6, IL-2, IL-7, and granulocyte colony-stimulating factor [1], while alveolar macrophages, CD138+ plasma cells, and T lymphocytes are profuse in bronchoalveolar lavage specimens [5].

In animal model studies and patients with ARDS, microparticles (MPs) are released into alveolar and vascular compartments [6]. These small vesicles contain membrane and cytosolic proteins, organelles, lipids, and RNA. MPs are shed from different cell types and then interact with other cells to provoke inflammation. In ARDS, released MPs are derived from endothelial and epithelial cells, neutrophils, monocytes, and lymphocytes [6]. Notably, lung microvascular endothelial cells express angiotensin-converting enzyme (ACE); endothelial cell-derived ACE+ MPs are prognostic for the development of ARDS in septic patients [7].

ACE2 appears to be the cellular receptor for SARS-CoV-2; Yan *et al*. [ 8] proposed that this coronavirus exploits ACE2 in host infection. ACE2 is a type I membrane protein normally expressed on endothelial and epithelial cells in lungs, heart, kidneys, and intestines [8]. In the endothelium, ACE2 maintains cellular homeostasis and function.

Enter “statins:” These 3-hydroxy-3-methylglutaryl-coenzyme A reductase inhibitors protect the heart, brain, and liver against post-ischemic injury by mechanisms that transcend lipid-lowering properties. Their mechanisms include stabilization of the vascular endothelium by enhancing endothelial nitric oxide synthase (eNOS) and ACE2 expression [9]. If endothelial-derived MPs are central to the pathogenesis of severe COVID-19 infection, there is scientific evidence that early administration of statins offers therapeutic efficacy in an organ system richly endowed with endothelial cells – the liver and its sinusoidal endothelial cells (SECs).

Atorvastatin injected intravenously (5 mg/kg body weight) 1 h before the onset of ischemia-reperfusion, a form of microvascular inflammatory liver injury that can complicate shock, conferred ~90% hepatoprotection [9] The mechanisms involve attenuation of systemic MP release, with diminished TNF-α, IL-6, MIP-1α, MCP-1, GM-CSF production, decreased thromboxane B2 production, vascular cell adhesion molecule-1 expression, and resultant abrogation of macrophage and neutrophil recruitment. Central to this protective effect of acute statin therapy was increased eNOS expression with enhanced eNOS activity, and protection of SECs observed directly by *in vivo* liver microcirculation studies [9]. Similar hepatoprotection by a dampening of the inflammatory response was observed in a different study using orally administered atorvastatin (5 mg/kg body weight) [ 10].

If SARS-CoV-2’s “hijack” of ACE2 is what initiates microvascular inflammation, leading to ARDS and multiorgan failure in fatal COVID-19 infections, statin intervention targeted early in the inflammatory cascade should ameliorate the detrimental pathogenic mechanisms that underlie ARDS, cytokine storm, and vascular collapse.

The utility of statins against ARDS is not new – it was tested in a prospective, randomized, double-blind, and placebo-controlled trial dubbed Heart Attack Research Program (HARP)-2 [11]. In patients with the hyperinflammatory subphenotype of ARDS (high IL-6 and soluble TNF-R-1), simvastatin (80 mg/day) administered enterally within 48 hours of lung injury, improved 28- and 90-day survival versus placebo [12,13]. Of note, simvastatin is not the most potent of statins and may not be well absorbed from the gastrointestinal tract of critically ill patients. Statins administered intravenously during coronary ischemia are more effective at decreasing infarct size than oral administration [14]. Utilized in other relevant pathophysiological vascular events such as acute coronary syndromes and stroke, statins have been shown to reduce serum CRP, systemic IL-1, IL-6, and TNF-α release [15,16].

We, therefore, propose a modification of the HARP-2 trial (registration: ISRCTN88244364) [11] using intravenous administration of a statin (e.g., atorvastatin, up to 5 mg/kg body weight) early in the course of suspected severe COVID-19 infections, ensued by follow-up intravenous or enteral statin up to 28 days post-randomization.

For entry into such a study, “severe” COVID-19 infection would be defined by the presence of the afore-mentioned poor prognostic factors on presentation and/or within 1 h of identification of acute lung injury. The primary outcome of such a trial, “Statin-HARP-2 plus 1” (nominally, “SHARP-3”), would be the number of ventilator-free days. The secondary outcomes would be lung and other organ function, 28- and 90-day mortality, safety, and laboratory analyses. The United Kingdom’s HARP-2 investigators stem from sites now dealing with overwhelming COVID-19 case burdens and high case fatality rates.

We believe “SHARP-3” merits a pilot clinical trial, early and pre-emptively in patients identified at presentation to be “at risk” of COVID-19 disease severity and mortality. If the preliminary findings prove promising, international multicenter trials can be rapidly initiated in countries still battling high mortality rates.
